# The Peritraumatic Behavior Questionnaire: development and initial validation of a new measure for combat-related peritraumatic reactions

**DOI:** 10.1186/1471-244X-13-9

**Published:** 2013-01-05

**Authors:** Agorastos Agorastos, William P Nash, Sarah Nunnink, Kate A Yurgil, Abigail Goldsmith, Brett T Litz, Heather Johnson, James B Lohr, Dewleen G Baker

**Affiliations:** 1 Veterans Affairs Center of Excellence for Stress and Mental Health, VA San Diego, CA, USA; 2 Department of Psychiatry and Psychotherapy, University Medical Center Hamburg-Eppendorf, Hamburg, Germany; 3 Department of Psychiatry, University of California, San Diego, CA, USA; 4 VA San Diego Healthcare System, San Diego, CA, USA; 5 Department of Psychiatry and Department of Psychology, Boston University School of Medicine, Boston, MA, USA; 6 National Center for Post Traumatic Stress Disorder, VA Boston Healthcare System, Boston, MA, USA; 7 Department of Psychiatry, University of California San Diego, 9500 Gilman Drive (0603V), 92093-0603V, La Jolla, CA, USA

**Keywords:** Peritraumatic behavior questionnaire, Peritraumatic dissociation, Peritraumatic reaction, Posttraumatic stress disorder (PTSD), Stress, Psychometric properties, Validity, Reliability, Screening, Assessment

## Abstract

**Background:**

Posttraumatic stress disorder (PTSD) is one of the most commonly observed stress-related conditions following combat exposure and its effective prevention is a high health-care priority. Reports of peritraumatic reactions have been shown to be highly associated with PTSD among combat exposed service members. However, existing instruments measuring peritraumatic symptoms were not specifically developed to assess combat-related peritraumatic stress and each demonstrates a different peritraumatic focus. We therefore developed the Peritraumatic Behavior Questionnaire (PBQ), a new military-specific rating scale focused upon the wide range of symptoms suggestive of combat-related peritraumatic distress in actively deployed Service Members. This study describes the development of the PBQ and reports on the psychometric properties of its self-rated version (PBQ-SR).

**Methods:**

688 Marine infantry service members were retrospectively assessed by the PBQ-SR within the scope of the Marine Resiliency Study after their deployment to war zone. Participants have been additionally assessed by a variety of questionnaires, as well as clinical interviews both pre and post-deployment.

**Results:**

The PBQ-SR demonstrated satisfactory internal consistency, convergent and discriminant validity, as well as high correlation with trait dissociation prior to deployment. Component analysis suggested a latent bi-dimensional structure separating a peritraumatic emotional distress and physical awareness factor. The PBQ-SR total score showed high correlation to general anxiety, depression, poorer general health and posttraumatic symptoms after deployment and remained a significant predictor of PTSD severity, after controlling for those measures. The suggested screening cut-off score of 12 points demonstrated satisfactory predictive power.

**Conclusions:**

This study confirms the ability of the PBQ-SR to unify the underlying peritraumatic symptom dimensions and reliably assess combat-related peritraumatic reaction as a general construct. The PBQ-SR demonstrated promise as a potential standard screening measure in military clinical practice, while It’s predictive power should be established in prospective studies.

## Background

Posttraumatic stress disorder (PTSD) is one of the most common stress-related conditions in active duty Service Members after combat exposure [1-3]. Early at-risk detection and prevention of the development of PTSD could therefore lead to substantial benefits concerning the potential personal, social, and economic consequences of this disorder in military populations [4-6]. Although prior literature has identified pre-existing and post-hoc risk factors for PTSD, limitations in the predictive value of these factors have recently prompted particular interest in the immediate peritraumatic response after trauma exposure as a fairly robust predictor of PTSD development.

Peritraumatic stress reactions refer to the different stress-associated behavioral, emotional, cognitive, and physiological symptoms during and immediately following a traumatic event (e.g., fear of dying, fear of losing emotional control, tachycardia, sweating, shaking, dizziness, dissociative symptoms, reduction of awareness, etc.). These reactions have demonstrated a strong and consistent association with the subsequent development of posttraumatic stress symptoms [7-18]. The degree of the peritraumatic adrenergic hyperactivation following a stressful or traumatic event is related to different patterns of physiological response [19], subjective experience of injury and trauma-related fears [11,20], resilience and coping mechanisms [21], poor military training outcomes [22,23], as well as symptomatic outcomes in trauma sequelae [21,24]. Pathophysiological pathways suggested for increasing the risk for PTSD include enhanced fear conditioning [25], behavioral avoidance coping [26], memory processing disruption [27-30], and over-consolidation of traumatic memories [31,32]. A prolonged continuation of the biological and behavioral responses following acute traumatic stress may lead to greater pairing of the traumatic memory with distress, initiating a cascade of secondary biological alterations with long-term adverse consequences including interference with fear extinction learning and retention [33-36]. Peritraumatic stress is, thus, a salient pre-clinical indicator of risk and could provide sufficient evidence to warrant evaluation, the provision of stress first aid, and, if necessary, sustained formal mental health intervention to promote healing and recovery [37-42].

Yet, the valid assessment of relevant behavioral, emotional, cognitive, and physiological information is the greatest challenge in combat-related, mental health research. Although instruments measuring peritraumatic symptoms exist, they were not specifically developed to assess combat-related peritraumatic reactions. In addition, the two most widely used peritraumatic scales each demonstrate different peritraumatic foci. The Peritraumatic Dissociative Experiences Questionnaire (PDEQ) mainly assesses dissociative symptoms and physical reactions, while the Peritraumatic Distress Inventory (PDI) primarily targets emotional/cognitive peritraumatic aspects [43-47]. Thus, when used independently, these scales may insufficiently capture the wide range of possible symptoms or reactions in the immediate aftermath of a combat-related trauma.

To redress this problem, we developed the Peritraumatic Behavior Questionnaire (PBQ). The goal was to generate a new, military-specific rating scale able to unify the underlying peritraumatic symptom dimensions and reliably assess combat-related peritraumatic reaction as a general construct in actively deployed Service Members. The goal of this primary study lies in the demonstration of satisfactory psychometric properties of the subjective PBQ version (PBQ - Self Report, PBQ-SR).

## Methods

### Subjects

All subjects enrolled were consenting active duty male Marines recruited from infantry battalions of the 1st Marine Division stationed at Marine Corps Air-Ground Combat Center, 29 Palms, or Camp Pendleton, in southern California. Because the MRS study targeted only ground combat units, no women were included in the study. Ethnic distribution was included in similar proportion to the representation in the Marine Corps [48] (cf. Table 1, legend). There were no other exclusion criteria. 706 deploying male Marines signed informed consent prior to deployment and were included in the study. Of all deployed participants, 688 Marines (mean age 22.1 ± 3.4 years) participated in post-deployment assessments, returned valid questionnaires and were included in our analysis (cf. Table 1). Of those, 404 Marine subjects reported no history of prior combat deployments.

**Table 1:** Means of psychometric scores assessed by different instruments in the sample

**Total scores**	**Whole sample**^**a**,**b**^**(n** = **688**^†^**)**		**No prior deployment**^**b **^**(n** = **404)**		**No prior deployment**^**b **^**and PBQ** > **12 (n** = **70)**	
	**Mean**	**SD**	**Mean**	**SD**	**Mean**	**SD**
**BAI** - **Post**-**deployment**	4.2	7.0	4.1	7.0	9.9	11.1
**BDI** - **Post**-**deployment**	4.7	6.8	4.6	7.0	11.4	11.3
**CAPS** - **Baseline**	7.3	12.7	7.2	12.7	13.8	18.0
**CAPS** - **Post**-**deployment**	10.6	16.0	9.7	15.4	22.7	25.4
**CAPS** - **Change**	3.1	16.9	2.5	17.2	8.7	26.5
**DES** - **Baseline**	9.2	10.8	10.0	11.6	16.2	15.7
**PCL** - **Baseline**	22.7	8.7	22.9	8.9	27.5	10.5
**PCL** - **Post**-**deployment**	23.0	9.1	22.5	8.4	30.5	13.9
**PCL** - **Change**	.3	9.8	-.4	9.7	3.0	13.6
**PDEQ** - **Post**-**deployment**	16.0	7.2	15.9	7.1	21.4	9.6
**PBQ** - **Total Score**	5.1	7.1	5.5	7.3	19.0	6.1
**SF12 Physical Score** - **Post**-**deployment**	53.6	6.54	54.0	6.3	52.6	8.4
**SF12 Mental Score** - **Post**-**deployment**	51.2	8.7	51.1	8.7	45.6	11.2
**PANAS**-**Positive Post**-**deployment**	33.9	9.0	34.1	9.1	34.5	9.4
**PANAS**-**Negative Post**-**deployment**	17.5	6.5	17.6	6.7	22. 5	7.4
**WHODAS Post**-**deployment**	13.9	4.2	13.9	4.4	17.0	7.2

Total scores of psychometric scores are reported as mean values ± SD. Measures were assessed either before (Baseline) or after deployment (Post-deployment). Changes indicate differences in the scores between pre and post-deployment. BAI: Beck’s Anxiety Index; BDI: Beck’s Depression Inventory-II; CAPS: Clinician Administered PTSD Scale; DES: Dissociative Experiences Scale-II; PANAS: Positive Affect Negative Affect Schedule; PDEQ: Peritraumatic Dissociative Experiences Questionnaire - Self Report; PCL: PTSD Checklist; SF-12: Short Form 12, Version 2; WHODAS: World Health Organization Disability Assessment Schedule II.

^a^ Demographics of whole sample in percentage (percentages under 1.0% are not reported).: Education (Some High School: 1.9%; GED: 1.5%; High School Diploma: 60.2%; Some College: 30.6%; Associates Degree: 2.5%; 4-year College Degree: 3.1%), Ethnicity (Not Hispanic or Latino: 74.6%; Mexican: 14.2%; Puerto Rican: 2.8%; South or Central American: 3.2%; Other Spanish culture or origin: 5.2%), Race (Black or African American: 4.9%; American Indian or Alaskan Native: 1.5%; Asian: 2.7%; Native Hawaiian or Pacific Islander: 1.0%; White: 83.7%; More than one: 7.2%).

^b^ There were no statistical significant differences in demographics between the different groups (data not shown).

^†^ Drop-out reasons included transfer, hospitalization, relocation or discharge of Service Members.

### Development of the peritraumatic behavior questionnaire

The goal was to develop a questionnaire that is easy to administer and score due to its simple rating and scoring instructions and clearly specified areas of evaluation. To develop the content for the Peritraumatic Behavior Questionnaire (PBQ) for use in a war zone, a panel of subject matter experts generated an initial pool of items and helped select the final set of items, emphasizing content validity [49,50]. The expert panel was formed by established clinical mental health professionals, with experience treating in-theater and post-deployment, combat-related, acute and posttraumatic stress symptoms, as well as research professionals with a focus in PTSD. The PBQ-SR was developed using the expert consensus method in four steps (Step 1: Item pool generation; Step 2: Item pool screening; Step 3: Items’ adjustment; Step 4: Final review) [51].

In step 1, a large item pool with different questions assessing peritraumatic reactions was generated. Constructs considered for inclusion were frequently observed peritraumatic symptoms seen in the battlefield of operations. Items were collected through summarizing clinical expertise derived from prior battle-field experience with traumatized Service Members, clinical notes and prior patients’ comments, a comprehensive literature review focused upon peritraumatic symptoms using a standard database search, and well established peritraumatic and dissociation questionnaires found to correlate with PTSD development, trauma history or symptom severity (e.g. Peritraumatic Distress Inventory – PDI; Peritraumatic Dissociative Experiences Questionnaire – PDEQ; Somatoform Dissociation Questionnaire - SDQ-20, Clinician Administered Dissociative Symptoms Scale - CADSS) [44,45,47,52-55]. In step 2, the item pool was screened to determine: (a) the questions’ applicability and relevance to military war zone experiences and culture and (b) the feasibility of objectively observing described symptoms, an attribute additionally rated by a focus group of Navy corpsmen previously deployed with Marine combat units. Peritraumatic reactions in battle are representative for extraordinary stress challenges for Service Members, who are faced with pervasive loss, life threat, extreme physical discomfort and moral conflicts, witnessing death and overcoming fear [56]. However during combat, the primary importance of these reactions lies in potential interference with maintenance of operational resilience, including mission effectiveness and optimal performance [57]. Thus as those aspects of peritraumatic reactions are already to deployed Service Members and salient to combat operations, special attention has been given to including such items in our questionnaire. Missing pool items addressing possible emotional changes or loss of control sometimes experienced in the aftermath of trauma or loss were identified and consequently also included into the item pool (i.e. items such as “acting inappropriately giddy or silly”; “uncontrollable laughing, crying, or screaming”; and “not feeling normally remorseful”). On the contrary, several questions reflecting ubiquitous experiences in theater (e.g., "I was afraid for my safety") were excluded from the item pool. Step 3 included slight language modifications of the collected questions in order to reflect military terminology and to qualify for use in military operational settings. In the final step, the selected items were reviewed again by the expert’s panel in order to ensure that all different battle-field related stress symptoms were adequately represented in the draft.

A total of 15 potential scale items were then selected from the remaining item pool (cf. Additional file 1). The final questionnaire items were refined and assembled into a 5-point-Likert scale structure, ranging from “Not at All True” to “Extremely True” (scored 0–4), to promote the comparability of our results to prior studies using existing questionnaires. Finally, the self-report version (PBQ-SR) was created by merely using first-person pronouns such as "I" and "my". All questions refer to symptoms during and/or immediately after the most stressful event experienced during the last deployment. It was further specified in the measure’s instructions that these reactions must have been unusual for the individual and not within their typical range of thoughts, emotions or behavior. The PBQ-SR can be completed within 5 min. The 15 individual items were summed to compute a single summary score. Higher scores indicate higher levels of peritraumatic symptoms.

### Methods

This study used data collected within the scope of the “Validation of the Peritraumatic Behavior Questionnaire” study, approved by the institutional review boards of the University of California San Diego, the VA San Diego Research Service (VA R&D and UCSD IRB approval #090563), the Brooke Army Medical Center and CENTCOM. This is a pilot study linked to the “Marine Resiliency Study” (MRS), a larger prospective investigation of active duty Marines approved by the institutional review boards of the University of California San Diego, VA San Diego Research Service, and Naval Health Research Center (VA R&D and UCSD IRB approval #070533) [48].

Informed consent for all deploying MRS Marines volunteering for this study was obtained prior to deployment. The PBQ-SR was administered to Marines at post-deployment assessment, along with other MRS study questionnaires, as part of the standard test battery for the MRS study. Exact post-deployment evaluation time periods varied between 8 and 12 weeks post-deployment depending on pre-scheduled assessment time points. Each platoon of each company were available for ½ day for collection of data, including paper-pencil questionnaires (approx. 1 ½ hrs). Two to four study staff members were specifically designated for monitoring of participants during questionnaires, as well as being present to answer questions, but were not constantly in the room where single participants filled out self-report measures. Survey response confidentiality was maintained at any time by providing spacing of at least 5 feet between chairs/desks where self-report questionnaires were completed by participants. Assessments were mainly conducted in Marine training spaces on a Marine Corps base, except for Marines not available to be tested with their units if discharged after deployment, transferred to a different unit or hospitalized. Those Marines were assessed at the VA San Diego Medical Center or at other locations.

### Measures

All MRS participants completed the following questionnaire prior to deployment only:

– The Dissociative Experiences Scale-II (DES): The DES is a 28-item, 11-point-Likert-scaled, self-report questionnaire assessing dissociative symptomatology during the past month. The DES has reported satisfactory psychometric properties, while higher scores indicate higher dissociative traits [58,59].

Participants also completed the following instruments both pre- and post-deployment:

– The PTSD Checklist (PCL): The PCL, a 17-item, 5-point-Likert-scaled, self-report questionnaire assessing PTSD symptom severity. The PCL has repeatedly demonstrated good psychometric qualities with higher scores indicating higher PTSD symptom severity [60,61]. In our study the PCL was used with respect to a specific traumatic event.

– The Clinician Administered PTSD Scale (CAPS): The CAPS was administered by trained clinicians in a face-to-face interview with MRS participants both pre- and post-deployment. The CAPS is a structured diagnostic interview that assesses the core and associated symptoms of PTSD and is considered the gold standard diagnostic manual for assessing PTSD in clinical research [62-64]. While several scoring rules and cut-off scores have been suggested, higher scores indicate higher symptom severity [64].

Finally, in addition to the PBQ-SR, the following questionnaires were administered after deployment only:

– Peritraumatic Dissociative Experiences Questionnaire – Self Report (PDEQ): The PDEQ is a 10-item, 5-point-Likert-scaled, self-report questionnaire assessing peritraumatic dissociation. The PDEQ has well-established psychometric properties, with higher total scores indicating increased peritraumatic dissociation [43,54] thus served as the reference standard in the evaluation of the PBQ-SR.

– Positive Affect Negative Affect Schedule (PANAS): The PANAS consists of two 10-item self-report scales designed to provide independent measures of positive (PANAS-P) and negative affectivity (PANAS-N). Each item is rated on a five-point-Likert scale, while low scores reflect the absence of the reported feelings [65,66].

– Beck Depression Inventory-II (BDI): The BDI is a 21-item, four-point-Likert-scaled, self-rating scale designed to measure emotional, cognitive, behavioural and somatic symptoms of depression over the previous two weeks and has become one of the most widely accepted instruments for detecting depression in normal populations [67,68].

– Beck Anxiety Index (BAI): The BAI is an established 21-item, four-point-Likert-scaled, self-report instrument assessing severity of anxiety symptoms during the past week [69].

– Short Form 12, Version 2 (SF-12): The SF-12 is a widely used and repeatedly validated, 12-item, 5 and 3-point-Likert-scaled, self-report questionnaire assessing health-related quality of life. Sub-scores are divided according to physical and mental health items. The SF-12 was developed as a more concise alternative to the original SF-36 and has been shown to mirror closely the physical and mental components of the original form, such as pain, general health, physical functioning, social functioning, vitality, etc. [70].

– World Health Organization Disability Assessment Schedule-II (WHODAS), Short Version: Developed from the original 36-item version, the WHODAS-II, Short Version is a 17-item, self-report generic health-status instrument assessing functioning in the last 30 days in six domains: communication, mobility, self-care, interpersonal, life activities, and participation [71]. The first 12 items are 5-point Likert-scale questions that assess areas of functioning while 5 additional items pertain to overall health, frequency of functional impairments, and interference caused by impairments. Only the 12 core items are aggregated into a total score.

### Statistical analysis

The main objective was the assessment of the major psychometric properties of the PBQ-SR. Internal consistency was assessed using corrected item-total correlations and Cronbach’s alpha coefficients. Construct validity was investigated by assessing the convergent and discriminant validity of the measure with respect to previously established measures. PBQ-SR internal consistency, as well as convergent and discriminant reliability were explored using the entire sample (N = 688). Concurrent validity of the PBQ-SR was assessed in order to explore its correlation to related measure scores assessed in our samples after deployment. In order to avoid bias of combat-related stresses or traumas associated with prior deployments, we excluded previously deployed Marines from this analysis. Concurrent validity correlations and cut-off scores were explored and calculated only in the sub-sample without any history of prior deployment (N = 404). A CAPS screening cut-off score of ≥45 was used to dichotomize the population, as suggested in the literature [64]. Changes in measures were computed as differences in total scores between pre- and post-deployment. Preliminary analyses were performed to ensure no violation of normality, linearity and homoscedasticity. Descriptive statistics are given in mean and standard deviation (SD) for ordinal scaled variables. Correlations are reported by the Pearson product–moment correlation coefficient and group differences were calculated by an independent-samples *t*-test. All tests of significance were 2-tailed, and p-values < .05 were considered significant. Statistical analyses were conducted using the Statistical Package for Social Sciences Version 20 (SPSS Inc., Chicago, IL).

## Results

### Clinical characteristics

The means and SD of all instruments assessed are presented in Table 1.

### Factor analysis

The 15 items of the PBQ-SR were subjected to principal components analysis (PCA) with direct oblimin rotation to investigate the underlying structure of the questionnaire. Prior to performing PCA, the suitability of data was confirmed (Kaiser-Meyer-Olkin value: .893; Barlett’s test of sphericity: p < .001). PCA suggested a two-factor solution with only two components exceeding an eigenvalue >1, explaining 38.1% and 12.4% of the variance respectively. An inspection of the screeplot revealed a clear break after the second component. The two-factor solution explained a total of 50.5% of the variance. The correlation between the two factors was *r* = .39. Aligned with the two-factor solution, we divided the two subscales of the PBQ-SR, entitled Emotional Distress Subscale (EDS) and Physical Awareness Subscale (PAS), according to the different item content of the components included. The EDS included emotional, perspective and coping items, while the PAS reflected awareness, dissociative and physical symptom items.

### Reliability

The PBQ-SR total score demonstrated good internal consistency, with a reported Cronbach’s alpha coefficient of .86. The inter-item correlation matrix revealed only positive values (mean .331), indicating proper scoring and that all items measured the same underlying attribute. The corrected item-total correlations varied between .406 and .644 for all items, while deletion of single items did not significantly change the alpha value, suggesting that the sum of the 15 items is acceptable to be used as a measure of peritraumatic reactions in battle-related trauma (cf. Table 2). The psychometric properties of both subscales were computed separately. The EDS and the PAS both displayed a good internal consistency (Cronbach’s alpha = 0.80 and .85 respectively).

**Table 2:** **Principal component analysis and convergent**, **discriminate and concurrent validity of the PBQ**-**SR**

**Item**	**Item**-**total correlation**	**α if Item deleted**	**Mean**	**SD**	**Pattern coefficients**		**Structure coefficients**		**Communalities**	**PDEQ**	**PANAS**-**P**	**CAPS**^†^
					**Component**		**Component**					
					**1**	**2**	**1**	**2**				
**PBQ 1**	.610	.851	.57	.953	.259	.**550**	.473	.**650**	.480	.347^**^	-.038	.415^**^
**PBQ 2**	.406	.866	.71	1.120	-.214	.**749**	.078	.**666**	.482	.144^**^	.052	.217^**^
**PBQ 3**	.516	.856	.30	.775	.021	.**669**	.281	.**677**	.459	.194^**^	-.005	.197^**^
**PBQ 4**	.586	.853	.58	1.085	-.095	.**832**	.228	.**795**	.640	.232^**^	.032	.286^**^
**PBQ 5**	.553	.857	.74	1.214	.061	.**667**	.321	.**691**	.481	.230^**^	.007	.287^**^
**PBQ 6**	.481	.858	.21	.631	.132	.**529**	.338	.**581**	.352	.215^**^	.027	.264^**^
**PBQ 7**	.546	.857	.15	.526	.**410**	.363	.**551**	.522	.415	.262^**^	-.009	.230^**^
**PBQ 8**	.628	.853	.21	.593	.**582**	.294	.**696**	.520	.558	.286^**^	-.046	.269^**^
**PBQ 9**	.644	.852	.21	.647	.**703**	.196	.**779**	.470	.640	.274^**^	.014	.251^**^
**PBQ 10**	.424	.861	.10	.466	.**785**	-.143	.**729**	.162	.549	.192^**^	.015	.120^*^
**PBQ 11**	.445	.861	.13	.477	.**785**	-.113	.**741**	.192	.560	.323^**^	.034	.201^**^
**PBQ 12**	.534	.857	.19	.589	.**581**	.184	.**653**	.410	.455	.309^**^	.028	.228^**^
**PBQ 13**	.453	.860	.15	.582	.**813**	-.124	.**765**	.192	.598	.280^**^	-.062	.249^**^
**PBQ 14**	.548	.855	.45	.948	.208	.**520**	.410	.**600**	.397	.258^**^	-.003	.275^**^
**PBQ 15**	.591	.852	.39	.889	.**591**	.228	.**680**	.458	.507	.399^**^	-.002	.364^**^
**PBQ Total**			5.10	7.08						.422^**^	.008	.436^**^
**EDS**										.391^**^	-.012	.309^**^
**PAS**										.325^**^	.017	.397^**^

Pattern and structure matrix of PCA with Oblimin rotation of two factor solution of PBQ-SR items and convergent, discriminate and concurrent validity of all items and total scores in the whole sample (n = 687). Correlations are given by the Pearson’s *r*. PBQ: Peritraumatic Behavior Questionnaire; EDS: Emotional Distress Subscale; PAS: Physical Awareness Subscale; CAPS: Clinician Administered PTSD Scale; DES: Dissociative Experiences Scale-II; PANAS-P: Positive Affect Negative Affect Schedule – Positive Affect Scale; PDEQ: Peritraumatic Dissociative Experiences Questionnaire – Self Report;.

^#^ Statistical trend: .05 < p < 0.1.

* p < .05.

** p < .001.

^†^ including only participants without prior deployment.

### Construct validity

Convergent validity was assessed using a Pearson product–moment correlation test to the PDEQ total score. We observed a robust statistically significant positive correlation between the total scores of the two measures (r = .422, p < .001, n = 667), as well as between each of the PBQ-SR items and PDEQ total score (cf. Table 2). Discriminant validity was examined by exploring the association between PBQ-SR total score and the divergent and theoretically unrelated constructs of PANAS-P scale. The PBQ-SR did not correlate with PANAS-P total score at all (r = .008, p = .846, n = 661). In the sample without prior deployment, PBQ-SR even showed a slight negative discriminant correlation to the PANAS-P, similarly to the PDEQ, confirming that the two scales are both equally unrelated to a conceptually different measure such the PANAS-P Scale. The EDS and the PAS both also displayed a satisfactory convergent and discriminant validity (cf. Table 2).

### Concurrent validity

Concurrent validity was measured by exploring the relation of the PBQ-SR total score to several scales assessing general anxiety, depression, negative affect, lower general health and posttraumatic symptoms after deployment and their changes over time, showing significant correlations (cf. Table 3). In addition, the PBQ-SR showed a significant positive correlation to the DES total score (r = .256; p < .001, n = 401; cf. Table 3). The same correlations of these measures to PDEQ were also computed and presented in Table 3. Controlling for CAPS and PCL baseline scores had no significant effect on our results. The EDS and the PAS both displayed a significant concurrent validity correlations to other measures (cf. Table 3).

**Table 3:** **Correlations of peritraumatic instruments to measures assessed post**-**deployment in participants without prior deployment** (**n** = **404**)

**Scales**	**PDEQ**	**PBQ**-**SR**	**EDS**	**PAS**
**BAI**	.348^**^	.478^**^	.442^**^	.385^**^
**BDI**	.381^**^	.531^**^	.402^**^	.487^**^
**DES**	.220^**^	.256^**^	.188^**^	.238^**^
**CAPS**	.457^**^	.400^**^	.305^**^	.361^**^
**CAPS** - **Change**	.185^**^	.201^**^	.200^**^	.148^*^
**PCL**	.485^**^	.488^**^	.395^**^	.423^**^
**PCL** - **Change**	.103^*^	.209^**^	.227^**^	.137^*^
**SF**-**12 Physical**	-.037	-.086^#^	-.143^*^	-.031
**SF**-**12 mental**	-.308^**^	-.336^**^	-.328^**^	-.261^**^
**WHODAS**	.266^**^	.372^**^	.384^**^	.276^**^
**PANAS** - **Negative**	.278^**^	.384^**^	.286^**^	.358^**^
**PANAS** - **Positive**	-.052	-.011	-.010	-.020

Correlations are given by the Pearson’s *r*. PBQ: Peritraumatic Behavior Questionnaire; EDS: Emotional Distress Subscale; PAS: Physical Awareness Subscale. BAI: Beck’s Anxiety Index; BDI: Beck’s Depression Inventory-II; CAPS: Clinician Administered PTSD Scale; DES: Dissociative Experiences Scale-II; PANAS: Positive Affect Negative Affect Schedule; PDEQ: Peritraumatic Dissociative Experiences Questionnaire – Self Report; PCL: PTSD Checklist; SF-12: Short Form 12, Version 2; WHODAS: World Health Organization Disability Assessment Schedule II. Change indicates differences in the scores between pre and post-deployment.

^#^ Statistical trend: .05 < p < 0.1.

* p < .05.

** p < .001.

### Multivariate analyses

Hierarchical multiple regression models were used to evaluate associations between PBQ-SR and PTSD symptomology while controlling for co-morbid anxiety and depressive symptoms, general health status, personality and emotional trait factors in terms of criterion validity. PTSD symptoms were indexed by CAPS and PCL total scores, however due to their high collinearity, CAPS and PCL total scores were entered as dependent variables in separate regression models. Preliminary analyses were conducted to ensure no violation of normality, linearity, multicollinearity and homoscedasticity. BDI, BAI, WHODAS, DES and PANAS-N total scores were entered as independent variables at step 1 and PBQ-SR in step 2. The final models were statistically significant and explained a total variance of 34.7% (F_(6, 389)_ = 34.4, p < .001) and 47% (F_(6, 389)_ = 57.6, p < .001) for CAPS and PCL scores, respectively. After controlling for the other measures, PBQ-SR remained a significant predictor of PTSD symptom severity as assessed by both measures (CAPS: β = .232; 95%-CI: .025 – .439; t = 2.2; p = .028; PCL: β = .181; 95%-CI: .079 – .283; t = 3.5; p = .001).

### Cut-off score

The relation between PTSD-risk status (CAPS score ≥ 45; whole sample: 4.8%, no prior deployment: 4%) and the PBQ total score was also examined using receiver operating characteristic (ROC) curves. Marines with a prior deployment were excluded from the analysis. The area under the curve was .85, which indicates that the PBQ is quite accurate in identifying PTSD-risk positive cases. The ROC diagram suggested a cut-off of 12 points (sensitivity 75.0%, specificity 85.6%) as the most appropriate one for screening purposes. In a direct univariate logistic regression, participants with a PBQ-SR total score above 12 had a 17.9 odds ratio of PTSD-risk group affiliation (*B* = 2.88; *SE* = .59; *Wald* = 23.5; *df* =1; *Sig.* <.001; *Exp(B)* = 17.9). Also after controlling for BDI, BAI, WHODAS and PANAS-N scores in a stepwise multiple linear regression model, a PBQ-SR score above 12 was the only significant predictor of affiliation in the PTSD-risk group (full model: *χ*^2^ = 46.2, p < .001, variance explained: 11.0 - 38.3%, PBQ-SR ≥12: *B* = 1.76; *SE* = .73; *Wald* = 5.9; *df* =1; *p* = .015; *Exp(B)* = 5.8), indicating that the cut-off score selected was able to distinguish between respondents. Using the PBQ-SR cut-off score of 12 points as a dichotomous variable, we compared the two resulting groups with respect to the other psychometric measures. Participants reporting PBQ-SR scores higher than the cut-off showed significantly higher anxiety (BAI: t_(399)_ = −5.075, p < .001), depression (BDI: t_(399)_ = −5.840, p < .001) and negative affect (PANAS-N: t_(399)_ = −6.117, p < .001) scores, lower general health scores (WHODAS: t_(399)_ = −4.171, p < .001) and a greater increase of PTSD symptoms after deployment (CAPS-Change: t_(395)_ = −2.232, p = .029; PCL-Change: t_(393)_ = −2.381, p = .020) than the control group.

## Discussion

This study validates a new instrument for the assessment of peritraumatic symptoms. To our knowledge, PBQ-SR is the first instrument specifically designed to measure several components of combat-related peritraumatic stress. The questionnaire was assessed using retrospective data on 688 Marine infantry service members with respect to the most stressful event during their last deployment. PBQ-SR demonstrated satisfactory psychometric properties with good internal consistency and discriminant validity as to PANAS-Positive Score. Descriptive analysis of each item, inter-item correlation and Cronbach's α stability after item deletion indicate that all 15 items of the questionnaire could be retained. The statistically significant positive correlation between both the PBQ-SR total score and all of its 15 items to the PDEQ confirms the ability of PBQ-SR to reliably assess peritraumatic reactions as a general construct.

Consistent with prior results research [46], there was a significant positive correlation between the PBQ-SR total score and measures of general anxiety, depression, negative affect and lower general health after deployment. PBQ-SR also showed high concurrent validity with respect to posttraumatic symptoms after deployment and their changes over time. PTSD severity strongly correlated not only with PBQ-SR total scores and the two subscales, but also with most of the fifteen individual PBQ-SR items. In order to compare the concurrent psychometric properties of PBQ-SR to PDEQ, we recalculated all correlations with respect to PDEQ (cf. Table 3). Our results suggest similar psychometric properties, while the PBQ-SR shows slightly better concurrent validity to almost every other measure assessed (cf. Table 3).

Linear and logistic regressions have shown that PBQ-SR total score remained a significant predictive factor of PTSD symptom severity, even after controlling for depression, general anxiety, negative affect and general health. Our results suggest a PBQ-SR cut-off score of 12 points for screening purposes. This score has been shown to correctly classify respondents at risk for PTSD even after controlling for other psychopathologies. In addition, participants exceeding this cut-off score also showed significantly higher anxiety, depression and negative affect scores, lower general health scores and a greater increase of PTSD symptoms after deployment when compared to the control group.

As literature suggests that peritraumatic symptoms partly rely on pre-existing factors, such as trait dissociation and may be an important risk factor for PTSD development and resilience [24,72,73], we also investigated the correlation between peritraumatic symptoms (PBQ-SR), trait dissociation assessed by the DES and posttraumatic symptoms (CAPS). Our results showed a statistically significant correlation between all three scores and confirm prior findings. However, even though the DES total score also correlated significantly with the CAPS total score (r = .188, n = 394, p < .001), the close correlation of PBQ-SR to the CAPS total score remained significant even after controlling for trait dissociation (r = .370, n = 393, p < .001).

A principal component analysis suggested the existence of two underlying factors: physical/dissociative (PAS) and perceptive/emotional (EDS) aspects of peritraumatic stress reaction. Both factors have demonstrated satisfactory convergent validity to PTSD specific measures and significant concurrent validity correlations to other measures such as general anxiety, depression, negative affect and general health (cf. Tables 2 and 3). At present, the two pre-existing scales assessing peritraumatic reactions (PDEQ and PDI) seem to each focus separately on different dimensions of peritraumatic symptoms. PDEQ’s underlying structure is shown to reflect more dissociative symptoms such as altered awareness and derealization (reflecting the PAS), while PDI focuses more on emotional peritraumatic distress symptoms (reflecting the EDS). Hence, our results suggest that PBQ-SR is a new scale that may have incremental validity by unifying and assessing the two major underlying peritraumatic symptom dimensions, which would otherwise require the administration of both PDEQ and the PDI. Assessing both dimensions also affirms the clinical relevancy of PBQ-SR and we therefore support the use of both subscales in clinical practice. The separate use of EDS and PAS could, however, be considered in psychiatric research.

In summary, our study replicates and extends the findings of other studies reporting peritraumatic dissociation to be a robust correlate of PTSD symptoms in adults. Our findings suggest that peritraumatic symptoms as measured by the PBQ may be useful indices of risk and need for early intervention for PTSD in service members deployed to combat zones, should, however, be prospectively validated through immediate peritraumatic assessment through in-theater administration of the PBQ-SR in the future.

### Limitations and strengths

Because the content development focused on the behavioral indicators of the phenomenology of service members under great strain in a war-zone, the PBQ stands out as a peritraumatic index uniquely suitable for military members. The PBQ is a questionnaire that is easy to administer and score due to its simple rating and scoring instructions and clearly specified areas of evaluation. The 5-point-Likert scale structure promotes the comparability of our results to prior studies using already existing scales. Concerning the psychometric properties of the PBQ-SR, excluding Marines with prior deployments from the predictive analyses has contributed to the reduction of bias risk due to prior stressors, while the use of the trauma-specific CAPS score likely contributed to a higher specific predictive power of our results. The size, as well as the homogeneity of our assessed sample (Marine, infantry) in terms of gender, age and kind of exposure type is a specific feature and further strength of our study, compared to validation studies designed for other scales. This may be an advantage for military-use of the questionnaire, but may limit its generalizability to other populations. In addition, as the optimal cutoff score was empirically established in our study, cross-validation in an independent sample appears necessary. Further limitations also include the lack of assessment of prior traumatic life events or childhood trauma, combat exposure level and test-retest variability of the measure. In addition, we evaluated the PBQ-SR on male Marine Corps infantry units who deployed to Afghanistan in a period of time that was relatively quiescent. It is an empirical question whether these results would be generalizable to more highly exposed troops, in other branches of service, and with women. However, the most significant limitation of the study is the retrospective, self-report assessment of peritraumatic symptoms, which precludes a conclusion on predictive validity of the PBQ-SR. Retrospective assessment of symptoms may also lead to distortion of recollections or bias due to current symptoms [74] and subjective assessment of combat-related symptoms could introduce bias and distortions related to cognitive barriers (i.e. fear of stigma, warrior ethos, criticism, fear of removal from unit, etc.) and adaptive denial coping mechanisms of Service Members [2,75]. Despite those concerns, our results suggest good reliability, validity and applicability of the PBQ-SR.

## Conclusion

The PBQ-SR is a reliable and valid new instrument for globally assessing different underlying dimensions of combat-related peritraumatic symptoms in active duty Service Members and demonstrates high correlation to various PTSD-specific, as well as related symptoms. The PBQ-SR may contribute to more accurate and earlier identification of individuals at high risk for developing combat-related acute and posttraumatic stress symptoms, allowing for further monitoring, coping support or immediate evidence-based treatment. The ability of the PBQ-SR to serve as a standard self-rated questionnaire with incremental validity in military clinical practice and predictive validity towards PTSD development, should, however, be prospectively validated through immediate, in-theater peritraumatic assessment in future studies.

The opinions and assertions contained herein are the private views of the authors, and do not necessarily reflect the official positions or policies of the Department of Defense, the Department of the Navy, or the Department of Veterans Affairs.

## Competing interests

All authors declare that they have no financial or non-financial competing interests.

## Authors' contributions

AA carried out all statistical analyses and contributed to the interpretation of data and drafting of the manuscript. WPN and DGB conceived of the study and have made substantial contributions to coordination and design. SN and KAY have been involved in drafting the manuscript and revising it critically for important intellectual content. AG, BTL, JBL and DGB contributed to the interpretation of data and have been involved in revising the manuscript critically for important intellectual content. HJ has made substantial contributions to acquisition of data. All authors have given final approval of the version to be published.

## Pre-publication history

The pre-publication history for this paper can be accessed here:

http://www.biomedcentral.com/1471-244X/13/9/prepub

## Supplementary Material

Additional file 1 The Peritraumatic Behavior Questionnaire – Self Report (PBQ-SR). Click here for file
