# Supplementary material for: The Peritraumatic Behavior Questionnaire: development and initial validation of a new measure for combat-related peritraumatic reactions
Source: BMC Psychiatry. 2013 Jan 5;13:9. doi: 10.1186/1471-244X-13-9 (PMC3598773; doi:10.1186/1471-244X-13-9)
Supplement: Additional file 1 — The Peritraumatic Behavior Questionnaire – Self Report (PBQ-SR). [file 1471-244X-13-9-S1.doc]

**Additional file 1** **The Peritraumatic Behavior Questionnaire – Self Report (PBQ-SR)**

|  | **Items** |
| --- | --- |
| 1 | For a period of time, I did not act like my normal self |
| 2 | For a period of time, I felt fearless and invulnerable, as if nothing could harm me |
| 3 | For a period of time, I did not care about my own or others’ welfare or safety |
| 4 | For a period of time, I felt no remorse for doing things that would have bothered me in the past |
| 5 | For a period of time, I was determined to get revenge |
| 6 | For a period of time, I was unable to stop laughing, crying, or screaming |
| 7 | For a period of time, I felt helpless and was unable to look out for my own welfare |
| 8 | For a period of time, I was confused and had difficulty making sense of what was happening |
| 9 | For a period of time, I was disoriented and was uncertain about where I was or what day or time it was |
| 10 | For a period of time, I could not move parts of my body |
| 11 | For a period of time, I froze or seemed to be moving very slowly, such that I could not do everything I wanted to do |
| 12 | For a period of time, my speech changed (such as stuttering, repeating words or phrases, or having a shaky or squeaky voice) |
| 13 | For a period of time, I was not able to fully carry out my duties (during or immediately after the event) |
| 14 | For a period of time, I believed I was going to die |
| 15 | For a period of time, I had an intense physical reaction such as sweating, shaking, or heart pounding |

All items were assessed by a Likert-scale instrument, with five possible responses ranging from “Not at All True” to “Extremely True” (scored 0–4). The questionnaires’ instructions specified to complete the items by circling the number that best describes the reactions experienced during and/or immediately after the most stressful event during the most recent deployment, but only if these reactions have been unusual and did not represent the normal way of thinking, feeling or behaving. Instructions: Please complete the items below by circling the number that best describes the reactions you experienced in yourself during and/or immediately after a recent stressful event. To apply to you, these reactions must have been unusual for you, and not the way you normally think, feel, or behave. If an item does not describe a change in your internal state or behavior during or after a stressful event, please fill in the circle for “not at all true.” Marked grey: Items composing the Emotional Distress Subscale (EDS); marked white: Items composing the Physical Awareness Subscale (PAS).
